# Vulvovaginal candidiasis: species distribution of *Candida* and their antifungal susceptibility pattern

**DOI:** 10.1186/s12905-018-0607-z

**Published:** 2018-06-15

**Authors:** Adane Bitew, Yeshiwork Abebaw

**Affiliations:** 1 Department of Medical Laboratory Sciences, College of Health Sciences, Addis Ababa University, P.O. Box1176, Addis Ababa, Ethiopia; 2 Department Clinical Laboratory, Fitche Hospital, P.O. Box 46, Oromia Administrative Region, Ethiopia

**Keywords:** Vulvovaginal candidiasis, Non-*albicans Candida* species antifungal drugs, Ethiopia

## Abstract

**Background:**

Vulvovaginal candidiasis is a global issue of concern due to its association with economic costs, sexually transmitted infections, and ascending genital tract infection. The aim of this study was to determine species distribution and antifungal susceptibility pattern of *Candida* species causing vulvovaginal candidiasis.

**Methods:**

A cross sectional study was conducted from November 2015 to December 2016 at the Family Guidance Association of Ethiopia. Vaginal swabs collected from study subjects that were clinically diagnosed with vulvovaginal candidiasis were cultured. Yeast identification and antifungal susceptibility testing were determined by the automated VITEK 2 compact system. The association of vulvovaginal candidiasis with possible risk factors was assessed and analyzed using SPSS version 20.

**Results:**

The overall prevalence of vulvovaginal candidiasis was 41.4%. The association of vulvovaginal candidiasis was statistically significant with previous genital tract infection (*p* = 0.004), number of life-time male sex partners (*p* = .037), and number of male sex partners in 12 month (*p* = 0.001). Of 87 *Candida* isolates recovered, 58.6% were *C. albicans* while 41.4% were non-*albicans Candida* species. The highest overall drug resistance rate of *Candida* species was observed against fluconazole (17.2%), followed by flycytosine (5.7%). All *Candida* isolates were 100% susceptible to voriconazole, caspofungin, and micafungin. *C. albicans*, was 100% susceptible to all drugs tested except fluconazole and flycytosine with a resistance rate of 2% each drug. *C. krusei,* was 100 and 33.3% resistant to fluconazole and flycytosine, respectively.

**Conclusions:**

High prevalence rate of vulvovaginal candidiasis and observation of high prevalence rate of non-*albicans Candida* species in the present study substantiate, the importance of conducting continuous epidemiological surveys to measure changes in species distribution from *C. albicans* to non-*albicans Candida* species in Ethiopia. Although, fluconazole still appeared to be active against all isolates of *C. albicans* and non-*albicans Candida* species high resistance rate of *C. krusei* against the drug may demonstrate a search for alternative antifungal drugs when treating vulvovaginal candidiasis caused by *C. krusei*.

## Background

Vulvovaginal candidiasis here is defined as isolation of *Candida* species in culture from study participants with sign and symptom of vaginal abnormalities. Vulvovaginal candidiasis, bacterial vaginosis, and trichomoniasis are the most common cause of vaginitis of which vulvovaginal candidiasis is the second most common after bacterial vaginosis [1, 2]. Nearly 5–10 million females seek gynecologic advice for vaginitis every year world-wide [3]. Many studies [3–8], have reported that three fourth (75%) of women will experience an episode of vulvovaginal candidiasis in their lifetimes, 50% of these will experience at least a second episode, and 5–10% of all women experience recurrent vulvovaginal candidiasis i.e., ≥4 episodes of vulvovaginal candidiasis per year.

Although the rate of vulvovaginal candidiasis is frequent, the reasons for its occurrence and recurrence are often unclear. Socio-demographic characteristics, use of antibiotics and oral contraceptives, diabetes mellitus, dietary practices, personal hygiene, sexual activity, and specific immunological defects have been identified as potential risk factors [3, 9–11]. However, the data supporting each of these factors are conflicting.

In most developing countries such as Ethiopia, vulvovaginal candidiasis is still received little attention since it is considered to be a trivial disease. However, vulvovaginal candidiasis has been identified as one of a global issues of concern due to its association with direct and indirect economic costs [12, 13], sexually transmitted infections, particularly HIV [14], and ascending genital tract infection [15].

Despite *Candida albicans* is the most common cause of vulvovaginal candidiasis, the frequency of vulvovaginal candidiasis caused by other *Candida* species, such as *C. tropicalis*, *C. glabrata*, and *C. krusei* is increasing, especially in HIV-infected women [16]. Yeast infections resistant to antifungal agents have been increasing and their frequency will likely continue to increase. Resistance to azoles antifungal therapy in *C. albicans* and non- *albicans Candida* species has become more common [17]. In Ethiopia, little is known regarding the distribution and the in vitro antifungal susceptibility profile of yeasts isolated from clinical samples including vaginal swabs. Poor laboratory set up and lack of expertise in the field can be incriminated as the major factors. In line with this, early and accurate identification of yeast pathogens and rapid antifungal susceptibility testing are the highest priority at least in cases of critical yeast infections. Against this background, the aim of this study was to identify and determine drug susceptibility profile of yeasts implicated in causing vulvovaginal candidiasis from patients attending at the Family Guidance Association of Ethiopian Addis Ababa model Clinic by employing the fully automated VITEK 2 compact system for the first time in Ethiopia. The VITEK 2 compact system is a fully automated and completely standardized microbiological system that performs both yeast identification and antifungal susceptibility testing simultaneously. A strong correlation between the VITEK 2 compact system in antifungal susceptibility testing and that of Clinical and Laboratory Standards Institute (CLSI) was demonstrated by Melhem et al. [18]. Apart from precise identification and susceptibility testing; shortened turnaround times, improved specimen handling, enhanced quality control, reproducibility and the ability to track results are further benefits of the system [19].

## Methods

### Study design, period and area

A cross sectional study was conducted from November 2015 to December 2016 at the Family Guidance Association of Ethiopian (FGAE) Addis Ababa model clinic. The association is the oldest and the largest non-government, not-for-profit organization with over 43 years of dedication in providing quality, broad ranging reproductive health services in Ethiopia complementing governmental efforts. It has 20 sexual and reproductive health care clinics across Ethiopia and 586 staff.

### Collection of socio-demographic, sexual behavior and reproductive health information

Socio-demographic, sexual behavior, and reproductive health characteristics such as age, education, marital status, number of life time male sex partner, history of abortion, previous history of genital tract infection, frequency of vaginal bathing, and frequency of changing underwear were collected by face- to- face interviews using a structured questionnaire. Age groups of patients were classified following WHO guideline [20].

### Specimen collection, inoculation, transportation and incubation

Prior to sample collection, patients with genital tract problem were clinically investigated by gynecologists on duty and signs and symptoms of vaginal abnormalities were recorded. Vaginal swabs were collected from study participants having different vaginal abnormalities such as vaginal discharge, itching, vulvar pruritus, irritation, pain during intercourse, and during urination using sterile rayon-tipped applicator stick swabs by experienced nurses. All vaginal swabs were then transferred without delay to the microbiology laboratory of the Department of Medical Laboratory Science, College of Health Sciences, Addis Ababa University. Each vaginal swab was inoculated onto to Sabouraud Dextrose Agar (SDA) supplemented with chloramphenicol (Oxoid, Basingstoke, UK) and incubated at 35–37°C for 18 to 24 h aerobically. Plates with no growth after 24 h were re-incubated for a further 24 h. Preparation and performance evaluation of culture media were done as per the instruction of the manufacturer.

### Yeast identification and determination of antifungal susceptibility

#### Inoculum size determination

Quality control yeast and pure cultures of yeast isolates were suspended in 3 ml of sterile saline (aqueous 0.45 to 0.50% NaCl, pH 4.5 to 7.0) in a 12 × 75 mm clear plastic (polystyrene) test tube to achieve a turbidity equivalent to that of a McFarland 0.50 standard (range, 1.80–2.20), as measured by the DensiChek (bioMe’rieux) turbidity meter. These suspensions were used for the inoculation of YST-21343 identification cards while AST-YS01 cards were inoculated after yeast suspensions were further diluted following the instruction of the manufacture.

#### Identification and determination of antimicrobial susceptibility

Species identification and antimicrobial susceptibility testing of yeasts were determined by the automated VITEK 2 compact system (bioMérieux, France) using YST-21343 and AST-YS01 cards. The system detects yeast growth and metabolic changes in the micro wells of thin plastic cards by using a fluorescence-based technology. In brief, identification cards were inoculated with quality control yeast and pure cultures of yeast isolate suspensions using an integrated vacuum apparatus. A test tube containing yeast suspension was placed into a special rack (cassette) and the identification card was placed in the neighboring slot while inserting the transfer tube into the corresponding suspension tube. The filled cassette was inserted manually into VITEK 2 compact reader-incubator module. After the vacuum was applied and air was re-introduced into the station, yeast suspension was forced through the transfer tube into micro-channels that fill all the test wells and inoculated cards were automatically sealed prior to loading into the carousel incubator. All card types were incubated automatically 35.5 ± 1.0 °C. Each card was removed from the carousel incubator once every 15 min, transported to the optical system for reaction readings, and then returned to the incubator until the next read time. Data were collected at 15-min intervals during the entire incubation period and final identification and antimicrobial testing (MIC and interpretation) results were obtained in approximately 18 h or less. A final identification of excellent, very good, good, acceptable, or low- discrimination was considered to be correct.

#### Quality control

Standard strains of *C. albicans* ATCC 10231 was used for quality control.

### Statistical analysis

All data from the investigation were coded, double-entered, and analyzed using SPSS version 20. Descriptive statistics and logistical regressions were used to estimate crude ratio with 95% confidence interval to the different variables. *P*-value < 0.05 was considered significant.

### Ethical clearance

All ethical considerations and obligations were duly addressed, and the study was conducted after the approval of the Department Research and Ethical Review Committee (DRERC) of the Department of Medical Laboratory Sciences, College of Health Sciences, and Addis Ababa University and the FGAE. Informed written consent was obtained from participants before data collection. Each respondent was given the right to refuse to take part in the study and to withdraw at any time during the study period. All information obtained from the study subjects were coded to maintain confidentially. When the participants were found to be positive for vulvovaginal candidiasis, they were informed by the hospital clinician and received proper treatment. An assent form was completed and signed by a family member and/or adult guardian for participants under the age of 18 years.

## Results

In the present study we assessed yeast carriage associated with vaginal candidiasis symptoms. The overall prevalence of vulvovaginal candidiasis was 41.4%. Subgroup prevalence of vulvovaginal candidiasis is presented in Table 1. Younger women, between 15 and 24 years had a somewhat lower prevalence (35.1%) of vulvovaginal candidiasis, while in the 25 years and older group, the prevalence was between 40.0 and 44.8%. The adjusted odds ratio showed that vulvovvaginitis was not significantly associated with age (*p* = 0.33).

**Table 1:** Prevalence of vulvovaginal candidiasis (culture positive) by selected characteristics in relation to demographic and sexual and reproductive health (210)

Characteristics	Number	Candidiasis		COR	95%Cl	P-value	AOR	95%Cl	P-value
		Yes	No						
Age in years									
15–24	54(25.7)	19(35.1)	34(62.9)	1			1		
25–44	116(55.2)	52(44.8)	65(56)	0.490	0.244–0.986	0.046	0.668	0.297–1.503	0.330
45–64	40(19)	16(40)	24(60)	0.348	0.147–0.822	0.016	0.479	0.177–1.299	0.148
Total	210(100)	87(41.4)	123(58.5)						
Marital status									
Un married	65(30.9)	27(41.5)	38(58.4)	1					
Married	107(50,9)	40(37.4)	67(62.6)	1.190	0.634–2.234	0.588			
Divorced	38(18)	20(52.6)	18(47.4)	0.639	0.286–1.431	0.277			
Total	210(100)	87(41.4)	123(58.5)						
Education									
Illiterate	47(22.3)	26(55.3)	21(44.7)	0.452	0.189–1.081	0.074	0.297	0.106–2.831	0.021
Primary school	59(28)	22(37.3)	37(62.7)	0.942	0.406–2.183	0.889	1.096	0.407–2.950	0.857
Secondary school	65(30.9)	25(38.5)	40(61.5)	0.896	0.393–2.041	0.794	1.313	0.521–3.305	0.563
College	39(18.6)	14(35.8)	25(64.1)	1			1		
Total	210(100)	87(41.4)	123(58.5)						
Number of life time male sex partner									
1–3	136(64.7)	48(35.2)	88(64.7)	1			1		
≥ 4	74(35.2)	39(52.7)	35(47.2)	2.043	1.148–3.635	0.015	0.469	0.230–0.956	0.037
Total	210(100)	87(41.4)	123(58.5)						
Number of male sex partner in 12 month									
0	28(13.3)	3(10.7)	25(89.2)	1			1		
1–2	129(61.4)	59(45.7)	70(54.2)	7.024	2.019–24.433	0.002	16.784	4.043–69.684	0.001
≥ 3	53(25.2)	25(47.1)	28(52.8)	7.440	2.001–27.669	0.003	14.988	3.454–65.030	0.001
Total	210(100)	87(41.4)	123(58.5)						
History of abortion									
Yes	52(24.7)	21(40.3)	31(59.6)	1.059	0.560–2.004	0.860			
No	158(75.2)	66(41.7)	92(58.2)	1					
Total	210(100)	87(41.4)	123(58.5)						
Previous BV/GTI									
Yes	95(45.2)	50(52.6)	45(47.3)	0.427	0.243–0.749	0.003	0.368	0.187–1.724	0.004
No	115(54.7)	37(32.1)	78(67.8)	1			1		
Total	210(100)	87(41.4)	123(58.5)						
Vaginal bathing /day									
1–3	128(60.9)	54(42.1)	74(57.8)	1					
≥ 4	82(39)	33(40.2)	49(59.7)	1.084	0.617–1.904	0.780			
Total	210(100)	87(41.4)	123(58.5)						
No of pants used in a day									
1–2 Pant for one day	92(43.8)	31(33.6)	61(66.3)	1			1		
One pant for 2–4 day	118(56)	56(47.4)	62(52.5)	0.563	0.320–0.988	0.045	0.507	0.245–1.013	0.045
Total	210(100)	87(41.4)	123(58.5)						

The prevalence of vulvovaginal candidiasis varied with education and marital status. Women who are illiterate were more affected than those patients with primary school education and above. Similarly, vulvovaginal candidiasis was higher among divorced study subjects (52.6%) compared to unmarried (41.5%) or married (37.4%) study subjects. Vulvovvaginitis also varied with selected reproductive health history characteristics. It was more in patients with previous genital tract infection (52.6%) than in those patients with no previous genital tract infections (32.1%). The adjusted odds ratio showed that vulvovaginal candidiasis was significantly associated with previous genital tract infection (*p* = 0.004). The magnitude of infection was more or less the same in study subjects with history of abortion and with no history of abortion. The magnitude of vulvovaginitis was statistically significant with the number of life-time male sex partners (*p* = .037) and number of male sex partners in 12 month (*p* = 0.001). The prevalence of vulvovaginal candidiasis was less than in those patients who changed underwear more frequently (two per day; 33.6%) than those who change their pants less frequently (one pant for 2–4 days; 47.4%). Similarly, patients who bathed their vaginal region more frequently were less affected than that did not bath their vaginal area more frequently (prevalence rate 40.2% versus 42.1%). As shown in Table 1, vulvovaginal candidiasis and personnel hygiene were not statistically associated.

Species distribution of *Candida* species is depicted in Table 2. A total of ten species of *Candida* were isolated from 210 women. *C. albicans* was the commonest isolate accounting for 58.6% of the total yeast isolate. The percentage of non-*albicans Candida* species (41.4%) was less than that of *C. albicans* (58.6%). Of the non-*albicans Candida* species, *C. krusei* was predominantly isolated (17.2%), whereas *C. lusitaniea and C. inconspicua* were found to be the least prevalent isolates (1.2%).

**Table 2:** Species distribution of *Candida* isolates from 210 patients with vulvovaginal candidiasis

Species of candida	Number of isolates	% of the total isolates
*C. albicans*	51	58.6
*C. krusei*	15	17.2
*C. dubliniesis*	8	9.2
*C. glabrata*	3	3.4
*C. inconspicua*	1	1.2
*C. tropicalis*	2	2.3
*C. kefyr*	2	2.3
*C. guillieromondii*	2	2.3
*C. lusitaniae*	1	1.2
*C. parapsilosis*	2	2.3
Total isolates 87		100

The overall drug susceptibility pattern of *Candia* species against the five antifungal drugs tested is shown in Table 3. The highest overall resistance rate of *Candida* species was observed against fluconazole (17.2%), followed by flycytosine (5.7%). All *Candida* isolates were 100% susceptible to voriconazole, caspofungin, and micafungin. As far as species specific antifungal resistance rates were concerned, *C. albicans*, the commonest isolate was 100% susceptible to all drugs tested except fluconazole and flycytosine with a resistance rate of 2% to each drugs. *C. krusei,* the second common isolate was 100 and 33.3% resistant against fluconazole and flycytosine, respectively.

**Table 3:** In vitro antifungal susceptibility pattern of the isolates (*n* = 87)

Species	Antifungal agent	MIC range (μg/ml)	% susceptible of the isolates	% intermediate of the isolates	% resistant of the isolates
*C. albicans (51)*	Fluconazole	≤ 1	98.0	2.0	0
	Voriconazole	≤ 0.12	100	0	0
	Caspofungin	≤ 0.25–0.5	100	0	0
	Micafungin	≤ 0.06 - ≤ 0.25	100	0	0
	Flycytosine	≤ 1	98.0	2.0	0
*C. krusei (15)*	Fluconazole	2 - ≥ 64	0	0	100
	Voriconazole	≤ 0.12 - ≤ 0.25	100	0	0
	Caspofungin	≤ 0.25–0.5	100	0	0
	Micafungin	≤0.12 - ≤ 0.25	100	0	0
	Flycytosine	≤0.25–16	66.7	0	33.3
*C. dubliniesis (8)*	Fluconazole	≤1–32	87.5	12.5	0
	Voriconazole	≤0.12	100	0	0
	Caspofungin	≤0.25	100	0	0
	Micafungin	≤ − 0.06–0.12	100	0	0
	Flycytosine	≤1–8	87.5	12.5	0
*C. glabrata (3)*	Fluconazole	4	100	0	0
	Voriconazole	≤0–1 2	100	0	0
	Caspofungin	≤0.25	100	0	0
	Micafungin	≤0.06	100	0	0
	Flycytosine	≤1	100	0	0
*C. tropicalis (2)*	Fluconazole	≤1	100	0	0
	Voriconazole	≤0.12	100	0	0
	Caspofungin	≤0.25	100	0	0
	Micafungin	≤0.06	100	0	0
	Flycytosine	≤1	100	0	0
*C. kefyr (2)*	Fluconazole	≤1	100	0	0
	Voriconazole	≤0.12	100	0	0
	Caspofungin	≤0.25	100	0	0
	Micafungin	0.12	100	0	0
	Flycytosine	≤1	100	0	0
*C. guillieromondii (2)*	Fluconazole	2	100	0	0
	Voriconazole	≤0.12	100	0	0
	Caspofungin	≤0.5	100	0	0
	Micafungin	0..5	100	0	0
	Flycytosine	≤1	100	0	0
*C. parapsilosis (2)*	Fluconazole	≤1	100	0	0
	Voriconazole	≤0.12	100	0	0
	Caspofungin	≤0.25	100	0	0
	Micafungin	≤0.25	100	0	0
	Flycytosine	≤1	100	0	0
*C. lusitaniae (1)*	Fluconazole	2	100	0	0
	Voriconazole	≤0.12	100	0	0
	Caspofungin	≤ 0.25	100	0	0
	Micafungin	0.5	100	0	0
	Flycytosine	≤1	100	0	0
*C. inconspicua (1)*	Fluconazole	≤1	100	0	0
	Voriconazole	≤0.12	100	0	0
	Caspofungin	≤0.25	100	0	0
	Micafungin	≤0.06	100	0	0
	Flycytosine	≤1	100	0	0

## Discussion

Information regarding the prevalence of vulvovaginal candidiasis in Ethiopia is not known. Regrettably, vulvovaginal candidiasis is not a reportable disease and the diseases is routinely diagnosed by sign and symptom without the support of laboratory diagnosis. As the result, the spectrum of yeasts implicated in causing the disease and their drug susceptibility profile is not known in the country. The prevalence of vulvovaginal candidiasis varies from one study to another. It is the second most common infection of the vulvovaginal area of symptomatic women accounting for about 17% to 42. % [2, 3, 9]. Although the prevalence rate of infection in our study (41.4%) was within the reported range, it was slightly higher than the prevalence rates reported by Ahmed et al. [3] and Olowe et al. [9], but lower than the prevalence rate reported by ERylander et al. [21]. Differences in socio-demographic characteristics, immune–status of patients [5], treating patients with broad spectrum antibiotics and immune suppressive drugs [22], and hormonal influences [23] have been identified as some of the factors for differences in the prevalence of the occurrence and/or recurrent vulvovaginal candidiasis among studies.

Age, level of education, and marital status as possible risk factors for vulvovaginitis were investigated in the present study. We did not find strong evidence about the associations between socio-demographic characteristics and the prevalence of vulvovaginal candidiasis. Among socio-demographic characteristics, age seems to be an important factor in the overall occurrence of vulvovaginal candidiasis. Out of 87 patients with vulvovaginal candidiasis, 71 (81.6%) patients were in their 2nd to 4th decade of life. Our result was comparable with previous study. Sobel et al. [11] indicated that vulvovaginal candidiasis is infrequent at puberty (the first occurrence of menstruation), but its frequency increases towards the end of the second decade of life (10–19 years of age) reaching its peak in the third (20–29 years of age) and fourth (30–39 years of age) decade of life. Even though the crude odds ratio revealed that the association of age and vulvovaginal candidiasis was statistically significant the association was not statistically significant as far as the adjusted odds ratio was considered. This may indicate that the association was influenced by other variables.

The infection was more in women that were illiterate than in those patients with primary school education and above, and the association of vulvovaginal candidiasis and level of education was statistically significant (*p* = 0.0.021). Improvement in personal hygiene and/or in economic status resulted from education may possibly explain the difference in the rate of infection between illiterates and those with better education. Our finding was consistent with the findings of Rathod et al. [24], but in contradiction with the conclusion reached by Vadav and Prakash [25]. Similarly, it was higher in divorced study subjects (52.6%) than unmarried (41.5%) or married (37.4%) study subjects. The association of vulvovaginitis with marital status was not statistically significant, but it was statistically associated with previous genital infection (*p* = 0.04). Our result was consistent with the findings of Rathod et al. [24].

Little attention has been given to reproductive health, behavioral factors, and personal hygiene as a risk factor for vulvovaginal candidiasis. In this study, the prevalence of vulvovaginal candidiasis by selected sexual behavior, reproductive health and personal hygiene was assessed. The results of the logistic regression analysis with the adjustment for potential confounders showed that vulvovaginal candidiasis was significantly associated with an increase in the number of life time male sex partner (*p =* 0.037) and male sex partners in 12 months (*p =* 0.001). Our finding was not in line with other previous reports. Sobe et al. [11] indicated that number of years women had been with their sex partners is not associated with vulvovaginal candidiasis. Furthermore, the role of frequency of coitus as a risk factor to vaginitis remains controversial [26]. The study of Janković et al. [27], showed that vulvovaginal candidiasis was statistically associated with continual wearing of panty liners and use of vaginal tampons during menstruation, a finding which is inconsistent with our result (*p* = 0.054). The association of the infection with the frequency of vaginal bathing was not statistically significant (*p* = .078).

Documented information regarding the spectrum and the in vitro antifungal susceptibility pattern of yeasts isolated from Ethiopian patients complaining of genital tract infection is not available. Among ten *Candida* species isolated in this study, the recovery rate for *C. albicans* was 51 (58.6%), 15 (17.2%) for *C. krusei*, 8 (9.2%) for *C. dubliniesis,* 3 (3.46%) for *C. glabrata,* 2 (2.3%) for each of *C. tropicalis, C. kefyr, C. parapsilosis,* and *C. guillieromondii* and 1 for each o*f C. lusitaniae and C. iconpspicua.* Our finding of *C. albicans* as the predominant species was consistent with similar earlier studies [11, 28]. Although numerous studies on the prevalence of different *Candida* species have led to the general agreement that *C. albicans* is the most commonly isolated species in patients with vulvovaginal candidiasis, there has been a growing trend of recovery of non-*albicans Candida* species. This is evident by the present study in which the isolation rate of non-*albicans Candida* species was 41.4%. Comparatively, less recovery rates of non-*albicans Candida* species of 31.7% in Belgium [29] and 19.8% in the United States [28] have been reported. Also comparatively higher recovery rates of 53.1, 65.0, and 57.5% non-*albicans Candida* species have been reported in studies conducted in India [3], Egypt [30], and Iran [31], respectively.

Differences in the recovery rate among non*-albicans Candida* species were observed between our study and many earlier studies. A recovery rate 14.3% for *C. glabrata*, 5.9% for *C. parapsilosis* and 8.0% for *C. tropicalis* was reported by Trama et al. [28]. The study of Sobel et al. [11], Nyirjesy [32], and Sobel et al. [33], revealed that *C. glabrata* was the predominant yeast among the non-*albicans Candida* species. The study of Bauters et al. [29] showed that *C. glabrata* as the most commonly isolated non*-albicans Candida* species (16.3%), followed by, *C. parapsilosis* (8.9%), *C. humicola* (1.6%), *C. krusei* (0.8%), and *C. lusitaniae* (0.8%). Hasanvand et al. [34] demonstrated that *C. albicans* as the most commonly isolated species followed by *C. glabrata, C. tropicalis*, and *C. parapsilosis.* In contrast to these reports, *C. krusei was* the dominant non-*albicans Candidia* species in the present study accounting for 17.2% of the total isolates. The significance of this finding could be explained with caution that *C. krusei* may replace *C. albicans* under selective pressure of fluconazole, resulting in infections refractory to the current fluconazole based treatment in Ethiopia. Like other African countries, the present guideline of the Ethiopian Ministry of Health for the management of candidiasis includes fluconazole as a first choice drug and ketoconazole and miconazole ointment as alternative antifungal agents [35, 36]. The widespread use of fluconazole or related azole antifungals to promote selection of resistant subpopulations by shifting colonization to more naturally resistant species especially *C. krusei* or *C. glabrata* was suggested by Alexander and Perfect [37]. Given that *C. glabrata* is naturally resistant to fluconazole, the rate of isolation of the yeast in the current study was lower than that of *C. krusei*. The spectrum and the relative frequencies of *Candida* species implicated in causing vulvovaginal candidiasis could probably vary from region to region and from country to country in the same region. Increased use of over-the-counter antifungal drugs inappropriately, frequently as a short, incomplete course of therapy, eliminating the more sensitive *C. albicans* and selecting for more azole-resistant non-*albicans Candida* species, prolonged therapy for recurrent candidiasis, and increased use of oral or topical azole agents—available as over-the counter have been suggested as a possible explanation for more frequent isolation of non-*albicans Candida* species from vulvovaginitis patients [38, 39].

The in vitro susceptibility testing of antifungal agents is becoming increasingly important because of the introduction of new antifungal agents and the recovery of clinical isolates that exhibit inherent or developed resistance to antifungal drugs. In this study, the drug susceptibility profile of all yeast isolates were tested against five antifungal drugs. Our study showed that fluconazole still appeared to be quite active against all isolates of *C. albicans* and non-*albicans Candida* species except for *C. krusei*. This indicated that there is no a continuing decline in the rate of fluconazole susceptibility, despite the continued widespread use of fluconazole both for therapy and for prevention for vulvovaginal candidiasis in Ethiopia. Our result was compatible with earlier studies [40, 41] that demonstrate the overall resistance in *Candida spp* to fluconazole and voriconazole has remained constant over a decade. Our result also invalidated, concerns about the rapid development of resistance to fluconazole after its introduction. In the present study, *C. krusei* which is reported to be intrinsically resistant to fluconazole [40] was 100% resistant to fluconazole. Therefore, our in vitro susceptibility results warrants clinicians working in health institutes with high proportion of cases of vulvovaginal candidiasis caused by *C. krusei*, which has high rates of resistance to fluconazole may consider the use of other alternative antifungal agents for treatment. The species was 100% susceptible to voriconazole. The voriconazole susceptibility of *C. krusei* vaginal isolates in the current study was consistent with other report [42]. We do not have an immediate explanation on the susceptibility difference between fluconazole and voriconazole against *C. krusei* as all azole antifungal drugs have a common mechanism of action, i.e., inhibition of ergosterol synthesis. [43]. Despite *C. glabrata* is reported to be naturally resistant species to fluconazole or related azole antifungals [37], all the three vaginal isolates of *C. glabrata* in the current study were susceptible to fluconazole and other azole antifungals. Our finding is more or less concurrent with the findings of Hasanvand et al. [34] in which out of 19 vaginal *C. glabrata* isolates only three isolates were resistant to fluconazole. Similarly, Richter et al. [39] documented that out of 112 isolates of *C. glabrata* only 67% (51.8% susceptible- dose- dependent, 15.2% resistant) was fluconazole non-susceptible. Discrepancies in the fluconazole susceptibility pattern in different studies should be verified by conducting further studies.

The in vitro susceptibility of all yeast isolates was 100% to both caspofungin and micafungin which block fungal cell wall synthesis by inhibiting the enzyme that synthesizes β-glucan. Similar result has been reported by Lyon et al. [40] and Pappas et al. [44]. In addition to this, the potency of both antifungal drugs was more or less the same against yeast isolates. Therefore, our study did not support disparity in potency (susceptibility) between the two echinocandins reported by Ostrosky-Zeichner et al. [45]. Ostrosky-Zeichner et al. [45], found that micafungin was 4 dilutions more potent than caspofungin. In contrast to azoles, echinocandins resistance does not seem to be a major concern, as global surveillance studies indicate that there has not been any significant epidemiological shift in the susceptibility of *Candida spp*. isolates to echinocandins [46]. Like-wise, almost all yeast isolates were susceptible to flucytosine with the exception of *C. krusei* which exhibited 33.3% resistant rate. About 2% of *C. albicans* and 12. 5% of *C. dubliniesis* were found out to be intermediate to flucytosine. Although, the prevalence of flucytosine resistance in yeast remains low, the speed at which yeast can develop resistance to flucytosine has driven clinicians to use the compound in combination with mainly amphotericin B [47].

## Conclusions

High prevalence rate of vulvovaginal candidiasis and observation of a high prevalence rate of non-*albicans Candida* species in the present study warrants, the importance of conducting continuous epidemiological surveys to measure changes in species distribution from *C. albicans* to non-*albicans Candida* species in Ethiopia. Although, fluconazole still appeared to be active against all isolates of *C. albicans* and non-*albicans Candida* species high resistance rate of *C. krusei* against the drug may support a search for alternative antifungal drugs when treating vulvovaginal candidiasis caused by *C. krusei*.
